# The Role of the VEGF-C/VEGFRs Axis in Tumor Progression and Therapy

**DOI:** 10.3390/ijms14010088

**Published:** 2012-12-20

**Authors:** Jui-Chieh Chen, Yi-Wen Chang, Chih-Chen Hong, Yang-Hao Yu, Jen-Liang Su

**Affiliations:** 1 Graduate Institute of Cancer Biology, College of Medicine, China Medical University, No. 91, Hsueh-Shih Road, Taichung 40402, Taiwan; E-Mails: d95442003@ntu.edu.tw (J.-C.C.); chihchenhong@gmail.com (C.-C.H.); 2 Graduate Institute of Biochemistry and Molecular Biology, National Yang-Ming University, No. 155, Sec. 2, Linong Street, Beitou District, Taipei 11221, Taiwan; E-Mail: bigheadfrog1@hotmail.com; 3 Department of Internal Medicine, Divisions of Pulmonary and Critical Care Medicine, China Medical University Hospital, No. 2, Yude Road, Taichung 40447, Taiwan; 4 Department of Biotechnology, Asia University, No. 500, Lioufeng Road, Wufeng Shiang, Taichung 41354, Taiwan; 5 Center for Molecular Medicine, China Medical University Hospital, No. 2, Yude Road, Taichung 40447, Taiwan

**Keywords:** VEGF-C, VEGFR-2, VEGFR-3, angiogenesis, lymphangiogenesis, metastasis

## Abstract

Vascular endothelial growth factor C (VEGF-C) has been identified as a multifaceted factor participating in the regulation of tumor angiogenesis and lymphangiogenesis. VEGF-C is not only expressed in endothelial cells, but also in tumor cells. VEGF-C signaling is important for progression of various cancer types through both VEGF receptor-2 (VEGFR-2) and VEGF receptor-3 (VEGFR-3). Likewise, both receptors are expressed mainly on endothelial cells, but also expressed in tumor cells. The dimeric VEGF-C undergoes a series of proteolytic cleavage steps that increase the protein binding affinity to VEGFR-3; however, only complete processing, removing both the *N*- and *C*-terminal propeptides, yields mature VEGF-C that can bind to VEGFR-2. The processed VEGF-C can bind and activate VEGFR-3 homodimers and VEGFR-2/VEGFR-3 heterodimers to elicit biological responses. High levels of VEGF-C expression and VEGF-C/VEGFRs signaling correlate significantly with poorer prognosis in a variety of malignancies. Therefore, the development of new drugs that selectively target the VEGF-C/VEGFRs axis seems to be an effective means to potentiate anti-tumor therapies in the future.

## 1. Characteristics of VEGF-C

Vascular endothelial growth factor (VEGF) was originally characterized as vascular permeability factor (VPF) because of its unique capability of inducing vascular leakage [1]. Shortly thereafter it was also independently described by Ferrara *et al.* as a selective inducer of mitogenic activity in endothelial cells [2]. The VEGF family has an integral role in many physiological and pathological processes including angiogenesis, lymphangiogenesis, vasculogenesis and vascular permeability [3,4].

To date, the VEGF family comprises seven members that include VEGF-A, VEGF-B, VEGF-C, VEGF-D, VEGF-E, VEGF-F and placental growth factor (PlGF). In mammals, the family consists of five members, each one encoded by a different gene [5]. The complexity is increased further by alternative splicing of VEGF-A, VEGF-B and PlGF, and proteolytic processing of VEGF-C and VEGF-D [6]. In addition, VEGF-E and VEGF-F are the VEGF homologues that exist in viruses and snake venom, respectively [7,8].

Among the growth factors, VEGF-C, due to its central roles in lymphangiogenesis and angiogenesis in embryos and tumors, is an important member of the VEGF family [9–13]. VEGF-C was identified in 1996 as a ligand for VEGF receptor-2 (VEGFR-2) and VEGF receptor-3 (VEGFR-3). These receptors are known as VEGFR-2, also called KDR (in humans) or Flk1 (in mice), and VEGFR-3 is also denoted as Flt-4. Binding of VEGF-C to VEGFR-2 or VEGFR-3 induces tyrosine autophosphorylation of the cytoplasmic tail of its receptors [14]. The VEGF-C gene has been identified on the Chromosome 4q34 in humans and Chromosome 8 in mice [15,16]. VEGF-C is comprised of over 40 kilobase pairs of genomic DNA and its coding sequence resides on all seven exons [17].

Nascent VEGF-C consists of a signal sequence, an *N*-terminal extension, the VEGF-homology domain, and a *C*-terminal extension that has cysteine-rich sequences resembling insect silk proteins [18]. After the signal sequence has been removed, two VEGF-C precursors are held together by intermolecular disulfide bonds to form an antiparallel homodimer. Subsequently, this dimer undergoes a series of proteolytic processes that increase the protein binding affinity to VEGFR-3 and VEGFR-2. The mature VEGF-C has been previously described as noncovalently linked homodimers [19]; however, more recent research has shown that VEGF-C also exists as covalently bound dimers. A structural study has shown that human VEGF-C is covalently linked by two disulfide bridges between Cys156 and Cys165 in the crystal structure [20]. Various studies have investigated the covalent dimerization of VEGF-C involved in binding to its receptors. A study generated VEGF-C point mutants by replacement of Cys with Ser indicating Cys165 but not Cys156 is involved in dimer formation, and Cys156 mutant efficiently binds VEGFR-3 but not VEGFR-2 [21]. However, the reported mutation of Cys156 into Ala in VEGF-C suggests that the ratio of monomeric molecules is increased resulting in the loss of binding to both VEGFR-2 and VEGFR-3 [22].

Besides in endothelial cells, VEGF-C has been detected in non-endothelial cells, including immune cells [23,24] and tumor cells. VEGF-C expression is closely related to lymphangiogenesis and lymphatic metastasis in a variety of human tumors [25–27]. Additionally, the effect of VEGF-C in liquid and solid tumors has also been reported to induce angiogenesis [28,29].

## 2. VEGF-C Signals via its Receptors

VEGFR-2 and VEGFR-3 are expressed prominently on vascular endothelial cells, but VEGFR-3 is also highly expressed on lymphatic endothelial cells (LECs). Recent studies, however, suggest that both receptors are also expressed in cancer cells and can contribute to tumor progression [30–32]. In response to their ligands, VEGFR-2 can modulate vascular endothelial survival, proliferation, migration and the formation of vascular tubes, while VEGFR-3 can promote the development of blood and lymphatic vasculature [3].

The VEGFRs have a similar organization; an extracellular domain composed of immunoglobulin (Ig)-like loops for ligand-binding, a transmembrane domain, a cytoplasmic juxtamembrane domain, a catalytic tyrosine kinase domain split by a kinase insertion domain and a *C*-terminal tail [33]. Previous studies have indicated that VEGF-C binding requires Ig-Loops 1 and 2 in VEGFR-3 [22], whereas binding to VEGFR-2 involves Loops 2 and 3 [20]. Since VEGF-C can bind to more than one receptor, this allows for the potential formation of receptor hetero- and homodimers [34,35]. Interestingly, VEGF-C can induce the formation and activation of VEGFR-3 homodimers and VEGFR-2/VEGFR-3 heterodimers, but not VEGFR-2 homodimers. VEGFR-2 homodimers, however, can be induced by VEGF-A [35].

For activation of the VEGFRs, in addition to dimerization, the precise orientation of receptor monomers is still required [36]. There are several studies describing how VEGFR-3 homodimers and VEGFR-2/VEGFR-3 heterodimers appear to be functionally distinct. VEGFR-3 homodimers are implicated in the three-dimensional organization of endothelial cells and lumen formation, while VEGFR-2/-3 heterodimers contribute to angiogenic sprouting [33,35,37]. A recent study showed that inhibition of receptor dimerization may exert a synergistic effect with antiangiogenic therapy using antibodies that block VEGFR ligand binding [38].

Numerous studies have reported the importance of VEGF-C/VEGFRs-mediated signaling in cancer progression. In human lung adenocarcinoma cells, VEGF-C triggers the activation of the Src-p38 mitogen-activated prtein kinases (MAPK) and subsequently up-regulates the expression of the transcription factor CCAAT/enhancer binding proteins (C/EBP), leading to the increased expression of contactin-1. Contactin-1, through the rearrangement of F-actin-containing microfilament bundles, plays an important role in the regulation of cancer metastasis [31]. In human acute myeloid leukemia (AML) cells, VEGF-C-induced expression of cyclooxygenase (COX)-2 is mediated via the VEGFR-3/JNK (c-Jun *N*-terminal kinase)/AP-1 pathway contributing to angiogenesis [29]. It was also shown that RNAi-induced knockdown of VEGF-C suppresses cell growth, invasion and migration in human non-small cell lung cancer (NSCLC) cells; moreover, down-regulation of VEGF-C is accompanied by decreased signaling of ERK, p38 and Akt pathways that are mediated via CXCR4, CCR7, VEGFR-2 and VEGFR-3 [39]. Taken together, VEGF-C signaling plays a pivotal role in the process of tumor development, which could be a target for therapeutic applications in cancer.

The lymphangiogenesis can also be caused by VEGF-C overexpression in the skin of transgenic mice [40]. Conversely, in transgenic mice, the overexpression of soluble VEGFR-3 can suppress lymphangiogenesis [41].

## 3. VEGF-C Is Involved in Regulating Tumor Lymphangiogenesis and Angiogenesis

VEGF-C, via VEGFR-3, is required for the sprouting of the initial lymphatic vessels in embryonic development [9,10]. VEGF-C also plays a crucial role in tumor lymphangiogenesis, which induces the formation of additional lymphatic vessels and provides routes by which tumors cells enhance metastasis to distant sites. In tumors, increased expression of VEGF-C also parallels an increase in lymph node metastasis, lymphatic invasion, distant metastasis, and poor prognosis [4,42]. Human breast carcinoma cells that overexpress VEGF-C were implanted orthotopically in the mammary fad pads of severe combined immunodeficiency (SCID) mice leading to the facilitation of tumor metastasis via the lymphatic vessels, but tumor spread can be inhibited by a soluble VEGFR-3 fusion protein [26]. In addition, a recent study also suggested that in mice bearing orthotopical xenografts with VEGF-C knockdown human lung carcinoma cells caused inhibition of lymphangiogenesis in tumor and surrounding tissues [43]. Indeed, utilizing high-resolution imaging demonstrates that VEGF-C promotes lymphatic metastasis by increasing the spread of cancer cells to lymph nodes [44].

Among the VEGFs, VEGF-A is widely studied and found to be responsible for angiogenesis while VEGF-C is thought to promote lymphangiogenesis. For example, transgenic mice overexpressing VEGF-C in the skin resulted in increased lymphatic vasculature, but the vascular structure was unaffected [40]. However, other data suggests that VEGF-C may be involved in the regulation of physiological and pathological angiogenesis in addition to lymphangiogenesis [13,45–47]. A recent study indicated the possibility that VEGF-C via VEGFR-2/VEGFR-3 heterodimers may actually induce angiogenic sprouts [35]. In addition, VEGF-C and its receptor VEGFR-3 are also related to angiogenesis in cancer [28,48]. Targeting VEGFR-3 may provide an alternative choice for anti-angiogenesis, particularly towards vessels that are present in resistance to VEGF or VEGFR-2 inhibitors therapy [49]. Analysis of the possible mechanism by which VEGF-C stimulates angiogenesis has been shown to be through a RhoA mediated pathway [50].

## 4. Regulation of VEGF-C Gene Expression

Increasing evidence has indicated that inflammation is closely associated with tumor progression and metastasis. For example, interleukin-6 (IL-6)-mediated signaling can regulate VEGF-C expression via the PI3K-Akt pathway, leading to lymphangiogenesis in human oral squamous cell carcinoma [51]. In non-small-cell lung cancer (NSCLC) cells, interleukin-17 (IL-17), a pro-inflammatory cytokine mainly secreted by activated T helper cells, can promote lymphangiogenesis via up-regulation of VEGF-C expression, in part through activation of the ERK 1/2 pathway [52].

Furthermore, if mutated, the activation of proto-oncogenes to oncogenes occurs in virtually all types of cancers, which have the potential to promote neoplastic transformation. A proto-oncogene protein Wnt1 can induce tumor growth and angiogenesis [53,54]. With regards to lymphangiogenesis, however, a recent study showed that Wnt1 could protect against melanoma progression by suppressing melanoma-derived VEGF-C expression, followed by reduced lymphangiogenesis and metastasis [55].

Tumor cells that interact with the extracellular matrix (ECM) are strongly implicated in tumor invasion and metastasis. Various studies have investigated the ECM and its related components, with regards to VEGF-C expression. Fibronectin (FN), an extracellular matrix cell-adhesive glycoprotein, is highly expressed in a variety of malignancies and appears to play important roles in the progression of metastatic disease. One of its alternative splicing domains, extra domain A (EDA), can promote the secretion of VEGF-C in colorectal cancer cells; a process related to the PI3K/Akt pathway [56]. Likewise, heparanase belongs to a kind of endoglycosidase, which is involved in the degradation and remodeling of the ECM via cleavage of the heparan sulfate [57]. A study suggested that heparanase activity is strongly implicated in tumor lymphangiogenesis and metastasis, which can be attributed, in part, to the induction of VEGF-C expression [58]. The human carcinoembryonic antigen-related cell adhesion molecule 1 (CEACAM1, also known as biliary glycoprotein or CD66a), can induce angiogenesis via increased expression of VEGF-C in bladder cancer cells [59].

In addition, growth factors, transcription factors and micro-RNAs might also be key players for the regulation of VEGF-C expression in cancer. Heregulin-β1 (HRG-β1), a member of the epidermal growth factor-like family, increases VEGF-C expression through the NF-κB-dependent signaling pathway in human breast cancer cells [60]. The lens epithelium-derived growth factor (LEDGF/p75), a member of the hepatoma-derived growth factor family, binds the VEGF-C promoter to increase VEGF-C expression and results in enhanced lymphangiogenesis and angiogenesis of ovarian carcinoma tumors [61]. SIX1, a homeodomain-containing transcription factor, is also involved in metastasis, particularly lymphatic metastasis, by enhancing VEGF-C expression in human breast cancer [62]. A recent report also showed that VEGF-C is significantly down-regulated by miR-1826 in human bladder cancer [63].

## 5. Modulation of VEGF-C/VEGFRs Signaling Axis

Co-receptors for VEGF-C, including the transmembrane neuropilin (Nrp) family can modulate downstream signal-transduction pathways of the VEGFRs. The exact signaling components downstream of VEGFRs by which Nrp regulates VEGF biology still remains to be elucidated. It has, however, been shown that Nrp1 forms a complex with VEGFR-2 in a ligand-specific manner to induce the activation of VEGF/VEGFR-mediated signaling [64,65]. There are two Nrp homologues, Nrp1 and Nrp2, both of which lack an intrinsic catalytic domain in the cytoplasmic tail. Nrp1 is largely expressed in arteries. Nrp2 expression is restricted to the lymphatic endothelium and is found at low levels in veins [66,67]. A previous study showed that Nrp1 is incapable of binding to VEGF-C [68]; however, other research has shown contradictory results. It has been shown that Nrp1 is able to bind VEGF-C [69]; however, Nrp1 is not necessary for VEGF-C-induced tumor lymphangiogenesis [70]. Accumulating evidence suggests that Nrp2 can bind to VEGF-C leading to activation of downstream signaling pathways involved in lymphangiogenesis [69–71]. Moreover, previous studies using co-immunoprecipitation techniques have shown that Nrp2 also interacts with VEGFR-2 and VEGFR-3 in the presence or absence of VEGF-C [69,72]. More recently, a study further demonstrated that treatment with anti-Nrp2 antibody results in a reduction of Nrp2/VEGF receptor complex formation [70]. These results indicated that an interaction of VEGF-C with VEGFR-2 or VEGFR-3 as well as neuropilins can be important for lymphangiogenesis and angiogenesis.

Additionally, previous studies have shown that heparan sulphate proteoglycans (HSPG) play a critical role in modulating tumor angiogenesis and lymph node metastasis [73,74]. A recent report has indicated that HSPG may be a novel co-receptor in VEGFR-3 activation by VEGF-C. Silencing lymphatic heparan sulfate chain biosynthesis reduced VEGF-C-mediated downstream ERK activation and inhibited VEGFR-3 receptor-dependent binding of VEGF-C to the lymphatic endothelial surface [75].

Recently, a soluble form of VEGFR-2 (sVEGFR-2) has been discovered, which is generated by alternative splicing [76]. The endogenous sVEGFR-2 exists as a monomer, which, unlike the dimeric transmembrane receptor, has poor affinity for VEGF-A [77]. Some studies indicate that sVEGFR-2 can bind to VEGF-C, thereby preventing its binding to VEGFR-3, consequently inhibiting lymphatic vessel growth [77,78]. Clinical reports have indicated that sVEGFR-2 levels are reduced in cancer patients and could be a potential biomarker for monitoring cancer progression [79–81].

## 6. Clinical Significance of VEGF-C Expression in Tumors

In acute myeloid leukemia (AML), increased expression of VEGF-C and VEGFR-3 in bone marrow samples was first reported by Fielder *et al.* [82]. High VEGF-C expressed levels may be an indicator for adverse prognosis and decreased drug responsiveness in patients with AML [83,84].

Bunone *et al.* reported that VEGF-C and VEGFR-3 are increased in neoplastic thyroid tissues, particularly in thyroid neoplasia that have lymph node metastases [85]. In addition, in papillary thyroid carcinomas (PTC), the most prevalent type of thyroid malignancy, a higher VEGF-C expression level is found in tumor tissues and the adjacent non-tumorigenic tissues, which is involved in lymph node metastasis and lymphovascular permeation [86]. Another study also found that increased serum VEGF-C levels were significantly correlated with nodal metastases and advanced tumor stages in PTC patients [87].

Coordinated expression of VEGF-C and VEGFR-3 in patients with non-small cell lung cancer (NSCLC) is an important influential factor in lymphatic metastasis; moreover, VEGF-C is expressed mainly in cancer cells and its receptor VEGFR-3 is predominantly localized in endothelial cells [88]. Another study addressed found that an increased ratio of VEGF-C and VEGFR-3 mRNA expression has a significant positive correlation with lymph node metastasis in NSCLC [89]. Furthermore, in NSCLC patients, the VEGF-C expression is significantly associated with the micro-lymphatic vessel density that correlates with poor survival and lymphangiogenesis [90].

The expression of VEGF-C in human prostate cancer also facilitates lymph node metastasis and tumor progression [91]. Previously, Tsurusaki *et al.* reported that VEGF-C mRNA expression was significantly higher in prostate cancer patients with lymph node metastases than those without. Moreover, an increased number of VEGFR-3-expressing vessels was observed in the stroma surrounding VEGF-C-positive tumors, suggesting that VEGF-C is implicated in prostate cancer progression [92]. VEGFR-3 expression is not limited to prostate carcinomas but is also found in normal prostate tissue and benign prostate hyperplasia. However, upregulation of VEGFR-3 is observed in prostatic carcinoma and is related to an increased risk of lymph node metastasis and recurrence [93,94].

Clinical significance of VEGF-C expression in gastrointestinal malignancy has also been reported [95]. Kitadai *et al.* were the first to exhibit the correlation between VEGF-C expression and clinicopathological features in human esophageal carcinoma. According to their study, VEGF-C is expressed by both carcinoma and stromal cells, and its expression level is related to advanced disease in human esophageal carcinoma [96]. Furthermore, in two histological types of esophageal tumors, squamous cell carcinoma and adenocarcinoma, high VEGF-C expression tends to correlate with poor survival in squamous cell cancer but not in adenocarcinoma of the esophagus [97]. The expression level of VEGF-C in the esophageal cancer tissue is markedly higher than in the corresponding non-cancerous mucosa. Clinical significance of high VEGF-C expression in patients with esophageal cancer is associated with lymph node metastasis and poor prognosis [98].

In the clinical specimens, the level of VEGF-C mRNA expression in gastric cancer is higher than in normal mucosa, which is closely associated with poorer prognosis [99]. VEGF-C expression at the tumor margin may be a sensitive marker for nodal metastasis, recurrence, and overall survival for patients with gastric carcinoma [100,101]. VEGF-C is detected mostly in the cytoplasm of cancer cells and VEGFR-3 is mainly distributed in the endothelium of lymphatic vessels. There is a trend for an increased frequency of VEGF-C and VEGFR-3 expression in gastric carcinoma tissues compared to normal gastric tissues, which is related to lymph node metastasis and low survival rates [102]. Additionally, VEGFR-3 has also been detected in gastric cancer cells suggesting that VEGF-C may directly stimulate tumor growth through both autocrine and paracrine manners [103].

In colorectal cancer, VEGF-C expression is also closely related to lymphatic involvement, lymph node metastasis, and depth of invasion [104]. The level of VEGF-C expression is significantly raised in colorectal cancer compared with polyps and normal mucosa. Furthermore, VEGF-C has been shown to correlate with advanced staged disease in colorectal cancer [105,106].

VEGF-C is overexpressed in breast cancer specimens as compared to adjacent normal mammary glands, which shows a significant correlation with lymphatic vessel invasion and survival rate [107–109]. Furthermore, nitric oxide (NO) may react with superoxide to generate the highly toxic peroxynitrite, which can react with proteins that form nitrotyrosine. High nitrotyrosine levels can also contribute to the induction of VEGF-C expression and lymph node metastasis, further leading to decreased freedom of disease and overall survival in patients with breast cancer [110].

In cervical carcinoma, high expression of VEGF-C is accompanied by increased expression of matrix metalloproteinase-2 (MMP-2), which is involved in tumor aggressiveness, resulting in poor prognosis and lower survival rates [111]. A study about the mechanism of how tumor cells enter the lymphatic system has also been provided. The high levels of VEGF-C expression, especially at the invasive edge of cervical cancers, may contribute to the high density of lymphatic vessels in peritumoral regions through lymphangiogenesis, which causes increased tumor aggression [112].

VEGF-C expression is found in ovarian carcinoma and its receptor VEGFR-3 is restricted to endothelial cells adjacent to tumor cells, which are closely associated with lymph node metastasis, peritoneal metastasis beyond the pelvis, and poor survival [113]. However, another survey provides inconsistent results, indicating that VEGF-C, VEGFR-2, and VEGFR-3 are expressed in both tumor cells and neighboring endothelial cells. The simultaneous expression of both VEGF-C and VEGFR-2 in tumor tissue is well correlated with tumor dissemination, such as peritoneal metastasis outside of the pelvis, lymph node metastases, and positive ascitic cytology. A higher positivity of VEGFR-3 is found in the tissue from metastatic ovarian cancer, although there is no statistically significant difference [114]. In addition, high-level expression of VEGF-C in ovarian cancer tissues could be involved in the more advanced clinical stages, which is probably due to the influence on MMP-2 expression, lymph vessel density, microvessel density, and low apoptotic index [115].

VEGF-C is implicated in the modulation of lymphangiogenesis and angiogenesis in bladder cancer, which is associated with lymph vessel density and microvessel density; leading to increased malignant potential of tumors and decreased patient survival [116].

## 7. Mechanisms of Action of VEGF-C Targeted Therapy

Due the role of VEGF-C/VEGFRs-mediated signaling in cancer progression and the observation that VEGF-C is highly expressed in a variety of malignancies, insights into the mechanisms related to the anti-tumor activity of VEGF-C targeted therapy might help improve current cancer therapy. The novel therapeutic strategies involved in VEGF-C signaling were (i) monoclonal antibodies; (ii) IgG fusion proteins or soluble receptor protein; (iii) multikinase inhibitors; and (iv) RNA interference.

### 7.1. Monoclonal Antibodies

In many tumors, high levels of VEGF-C expression have been correlated with lymphatic metastasis and overall poor prognosis. VEGF-C is therefore an attractive target for cancer therapy using an anti-VEGF-C antibody to prevent disease progression. Recently, a study using antibody phage-display to develop a human monoclonal antibody fragment that exhibits a high affinity and specificity for the mature form of human VEGF-C has been published, but treatment efficiency for tumors has yet to be elucidated [117].

VEGF-C exerts its action by binding to its corresponding receptors. VEGFR-3 is a specific receptor for VEGF-C; however, proteolytically processed VEGF-C also allows binding to VEGFR-2. A previous study has employed receptor-specific antagonist antibodies to improve cancer outcomes in an orthotopic mouse model of spontaneous breast cancer metastasis. Reduction of regional and distant metastases by inhibition of VEGFR-3 activation is more efficient than inactivation of VEGFR-2. However, VEGFR-2 blockade mainly tends to inhibit tumor growth and angiogenesis rather than metastasis. In addition, combination therapy with the anti-VEGFR-2 and anti-VEGFR-3 blocking antibodies diminishes metastases with a greater effect than either antibody alone [118]. Another study showed that in subcutaneous and orthotopic human prostate tumor xenograft models, abrogation of VEGFR3 signaling significantly inhibited lymph node metastasis, but not growth of the prostatic tumor, whereas blockade of VEGFR-2 signaling efficiently repressed tumor blood vessel density and tumor growth without a corresponding reduction in nodal metastasis [119].

Typically receptor-blocking antibodies mainly targeted the ligand-binding domains of VEGFR; moreover, a recent study reported on antibodies directed against the extracellular domain of VEGFR-3 inhibiting receptor dimerization. The results from this report showed that the combined use of antibodies blocking both the ligand binding and the receptor dimerization improves therapeutic outcomes compared to either antibody alone [38].

VEGF-C also binds to its co-receptor, Nrp-2, which modulates developmental lymphangiogenesis and tumor metastasis. Blocking VEGF-C binding to Nrp-2 by treatment with anti-Nrp2 antibody was demonstrated. Anti-Nrp-2-specific antibody could modulate lymphatic endothelial cell migration *in vitro*, reduce tumoral lymphangiogenesis and metastasis *in vivo* [70].

### 7.2. IgG Fusion Proteins or Soluble Receptor Protein

A soluble VEGF-C competitor, named sVEGFR3-Fc, was addressed for trapping VEGF-C to reduce tumor metastasis. Mice were implanted s.c. with tumors derived from metastatic cancer cells expressing high levels of VEGF-C leading to a significantly higher incidence of lymph node metastases. This phenomenon was inhibited when sVEGFR3-Fc was produced using an *in vivo* gene delivery by adeno-associated virus [120]. Other investigators found similar results. First, they created sVEGFR-3 overexpressing human prostate cancer cell lines using lentiviral vectors. Then mice bearing sVEGFR-3 overexpressing tumors revealed a decrease in tumor growth, blood vasculature, and metastasis to regional lymph nodes [119]. There has been recent progress in the development of a new receptor-immunoglobulin (Ig) fusion protein, which could bind VEGF-A and VEGF-C simultaneously. This fusion protein was shown to effectively inhibit tumor growth and metastasis [121]. In addition, the soluble form of VEGFR-2 (sVEGFR-2), which has recently been described as an endogenous inhibitor of lymphangiogenesis, may be developed for the treatment of cancer [80].

### 7.3. Multikinase Inhibitors

Binding of VEGF-C to VEGFRs, leading to kinase phosphorylation and activation has a vital role in lymphangiogenesis and tumor metastasis. Small molecule tyrosine kinase inhibitors have a variety of adverse side effects; however, they also offer the potential to inhibit several kinases and impede tumor growth and metastasis by regulating multiple mechanisms to prolong overall and progression-free survival.

Cediranib, a potent inhibitor of VEGFR tyrosine kinases, is capable of simultaneously inhibiting the activity of VEGFR-2 and VEGFR-3 resulting in suppression of angiogenesis and lymphangiogenesis [122]. Multi-kinase inhibitor E7080, a potent inhibitor of both VEGFR-2 and VEGFR-3 kinase, effectively prevented regional lymph node metastases and further tumor growth [123]. Multi-kinase inhibitor Sunitinib can block both VEGFR-2 and VEGFR-3 phosphorylation and abrogate the activation of the downstream molecules ERK1/2 and Akt, which attenuates cell proliferation, migration and tube formation under VEGF-C stimulation [124].

### 7.4. RNA Interference

In recent years, RNA interference (RNAi) has emerged as a powerful tool to effectively silence specific genes, which holds great promise with regard to cancer therapy [125]. Accumulating evidence reveals that RNA-mediated knockdown of VEGF-C results in a significant inhibition of cancer progression [39,63,126]. However, simultaneous silencing of VEGF-A and VEGF-C decreased lymph node and lung metastasis, rendering this combined therapy to be more effective than either alone [127]. Furthermore, silencing the expression of VEGFR-2 or VEGFR-3 has also been proposed to be an effective method to reduce the metastatic potential [128,129].

## 8. Conclusions

**Figure 1:**
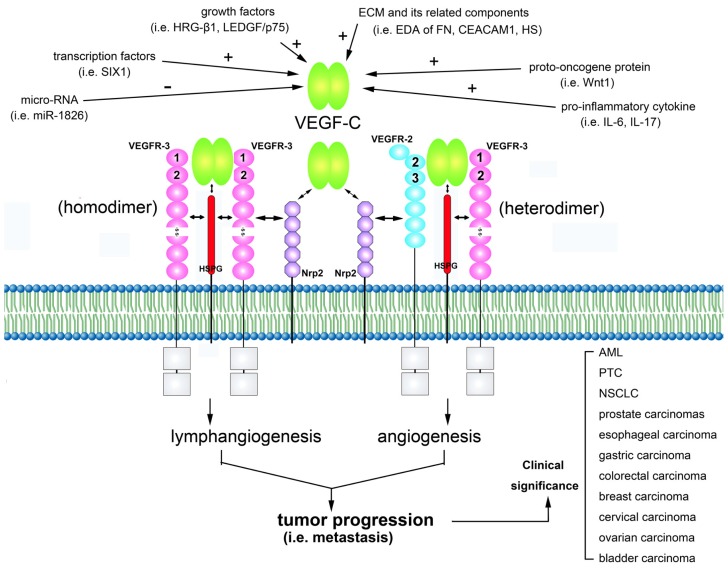
Schematic outline of the interactions of vascular endothelial growth factor C (VEGF-C) with its receptors and co-receptors leading to biological effects in tumor progression. VEGF-C can be proteolytically processed, and the mature form allows binding to VEGF receptor-2 (VEGFR-2) and increased affinity for VEGFR-3. VEGF-C expression is modulated by different manners. Binding of the dimeric VEGF-C stimulates receptor dimerization, leading to the formation of VEGFR-3/VEGFR-3 homodimers and VEGFR-2/VEGFR-3 heterodimers, which is implicated in lymphangiogenesis and angiogenesis, respectively. VEGFR Ig-like domains are involved in VEGF binding as indicated by the loop numbers in the figure. The complex network of intracellular signal transduction pathways results in tumor progression. The clinical significance of the VEGF-C/VEGFRs axes has been described in a variety of malignancies.

The VEGF-C signaling through both VEGFR-2 and VEGFR-3 plays a critical role in cancer progression by regulating lymphangiogenesis and angiogenesis. High-level activation of the VEGF-C/VEGFRs axis correlates with increased invasion and metastasis in various malignancies (Figure 1). The development of new drugs that hinder VEGF-C signaling could provide an effective means to potentiate anti-tumor therapies in the future. Results from clinical/preclinical development have led to the clinical approval of potential novel targets for antiangiogenic and anti-lymphangiogenic therapies [130]. Clinical studies to test these novel therapies are ongoing [131] Here, we review the recent progress in understanding the role of the signaling mechanisms of the VEGF-C/VEGFRs axis in the regulation of the biological properties and have the clear prospect of being able to utilize this knowledge translationally for novel treatment strategies for the deadliest aspects of cancer.
